# Mirror-touch and ticker tape experiences in synesthesia

**DOI:** 10.3389/fpsyg.2013.00776

**Published:** 2013-11-07

**Authors:** Charlotte A. Chun, Jean-Michel Hupé

**Affiliations:** Centre de Recherche Cerveau et Cognition, Université de Toulouse and Centre National de la Recherche ScientifiqueToulouse, France

**Keywords:** synaesthesia, subjective experience, phenomenology, grapheme color, number lines, spatial forms

## Abstract

A fundamental question in the field of synesthesia is whether it is associated with other cognitive phenomena. The current study examined synesthesia's connections with phenomenal traits of mirror-touch and ticker tape experiences, as well as the representation of the three phenomena in the population, across gender and domain of work/study. Mirror-touch is the automatic, involuntary experience of tactile sensation on one's own body when others are being touched. For example, seeing another person's arm being stroked can evoke physical touch sensation on one's own arm. Ticker tape is the automatic visualization of spoken words or thoughts, such as a teleprompter. For example, when spoken to, a ticker taper might see mentally the spoken words displayed in front of his face or as coming out of the speaker's mouth. To explore synesthesia's associations with these phenomena, a diverse group (*n* = 3743) was systematically recruited from eight universities and one public museum in France to complete an online screening. Of the 1017 eligible respondents, synesthetes (across all subtypes) reported higher rates of mirror-touch and ticker tape than non-synesthetes, suggesting that synesthesia is associated with these phenomenal traits. However, effect sizes were small and we could not rule out that response bias influenced these associations. Mirror-touch and ticker tape were independent. No differences were found across gender or domain of work and study in prevalence of synesthesia, mirror-touch or ticker tape. The prevalence of ticker tape, unknown so far, was estimated at about 7%, an intermediate rate between estimates of grapheme-color (2–4%) and sequence-space synesthesia (9–14%). Within synesthesia, grapheme-personification, also called ordinal-linguistic personification (OLP) was the most common subtype and was estimated around 12%. Co-occurences of the different types of synesthesia were higher than chance, though at the level of small effect sizes.

## Introduction

When probing atypical subjective experiences, for example when asking people questions such as, “Do numbers have colors?” the most typical reaction from people who do not have such experience is puzzlement. Those who do may also be puzzled, either by the idea that not everyone shares this experience or, on the contrary, by the discovery that they are not unique. The more we ask questions about the intimacy of subjective experience, the greater diversity of responses we seem to get. Is there any “normal” or at least common subjective experience? Synesthesia, which at first seemed a very rare and extraordinary condition, now seems to be shared by a large fraction of the population. As soon as researchers started considering so-called atypical subjective experiences, the social demand for numbers has been high, and quite legitimately: people suddenly either discover that they are “different” or may take comfort from not being that “weird.” So the first question is how normative this experience is. Only a few large-scale, systematic studies have been able to provide prevalence estimates so far. The present study aims to contribute to this endeavor by including many subtypes of synesthesia as well as two other, possibly related, subjective phenomena: mirror-touch and ticker tape.

We consider synesthesia as the subjective phenomenon of additional experiences that sometimes, but not always, involves mixing sensory modalities: perceptual, emotional, or imaginary stimulation evokes sensory, representational, cognitive, or affective “synesthetic” experiences. These associations are supplementary, automatic, idiosyncratic, arbitrary, and involuntary (Hupé et al., 2012; Simner, 2012). Though some common trends in synesthetic pairing have been identified (e.g., light colors with high-pitched notes; Ward et al., 2006; common letter-color combinations; Rich et al., 2005; Simner et al., 2005), specific synesthetic associations are distinctive to an individual. A common example is grapheme-color synesthesia, in which letters or numbers evoke color associations (i.e., 7 is green).

Synesthesia runs in families (e.g., Barnett et al., 2008) and there is evidence of genetic influence on its development (Asher et al., 2009; Tomson et al., 2011). However, environmental factors also play a role in the expression of synesthesia, evidenced by: (1) variation in synesthetic subtypes and specific associations within families (Barnett et al., 2008) and (2) examples such as lexical-gustatory synesthetes associating words with foods they ate during childhood (i.e., British synesthetes tend to associate words with flavors like jam and not with chili pepper or wine, which are rarely consumed during childhood; Ward and Simner, 2003) or some grapheme-color synesthetes whose associations correspond to the colored letters from their childhood toys (Witthoft and Winawer, 2013).

Estimates of the prevalence of synesthesia vary depending on the methodology and criteria employed. A large-scale, systematic study including letter-color, number-color, month-color, day-color, word-color, person-color, person-smell, taste-shape, and music-color indicated a prevalence of 4.4% in the Scottish population (*n* = 500; Simner et al., 2006). However, this study did not include the two other most common forms of synesthesia (according to Flournoy, 1893), sequence-space synesthesia, a visuospatial representation of sequences, such as numbers (“number forms”: Galton, 1880a,b), and grapheme-personification synesthesia, also named ordinal-linguistic personification (OLP): the association of characteristics, such as gender and personality, with linguistic sequences (Simner and Holenstein, 2007). Moreover, this prevalence value was based on the number of synesthetes confirmed with objective measures (Simner et al., 2006), aiming to estimate the lower bound, not the upper bound of the proportion of synesthetes.

Mirror-touch is the automatic, involuntary experience of tactile sensation on one's own body when others are being touched (Blakemore et al., 2005). For example, watching another person's arm being stroked can evoke a physical sensation of the touch on one's own arm. This phenomenon is proposed to arise in part from atypical representations of self-other discrimination (Banissy and Ward, 2007, 2013). Banissy et al. (2009) distinguished between specular subtype (mirrored sensations) and anatomical subtype (non-mirrored sensations, felt on the same side of the body as the true touch), finding that specular was more common (*n* = 17/21).

About 10.8% of an undergraduate British sample (*n* = 567) reported experiencing mirror-touch. Further interview of these 61 subjects inquiring about the location and description of tactile sensations during video observation of touch reduced the number of subjects with potential mirror-touch to 2.5% (*n* = 14). The prevalence of mirror-touch was further estimated from this sample, identifying only 9 subjects who showed Stroop-like effects stronger than controls in a tactile-congruency paradigm (Banissy et al., 2009). However, synesthetic Stroop-like effects can be elicited in non-synesthetes trained to learn grapheme-color associations (e.g., Elias et al., 2003; Meier and Rothen, 2009) and can be mild or absent in synesthetes verified with consistency tests (Hupé et al., 2012; Ruiz and Hupé, under review). Therefore, Stroop interferences likely measure the strength more than the authenticity of phenomenal associations. Nonetheless, the conservative prevalence estimate of 1.6% using this paradigm suggests that mirror-touch is at least as common as grapheme-color synesthesia in the British population, also using stringent criteria (Simner et al., 2006). The intermediate estimate of 2.5% highlights the potential for misunderstanding or false report inherent in brief self-report measures (Banissy et al., 2009).

Ticker tape experiences are the automatic visualization of words as they are thought or spoken, often seen in the mind's eye as static subtitles or a dynamic teleprompter (Galton, 1883,^1^; Day, 2005). For example, when being spoken to, a ticker taper might see mentally the words as they exit the speaker's mouth. To our knowledge, there are no prevalence estimates available for ticker tape experiences, so the present study may be the first one to report on this prevalence.

Mirror-touch and ticker tape experiences share some commonalities with synesthesia, and could therefore be considered as subtypes of synesthesia (e.g., Serino et al., 2008; Fitzgibbon et al., 2010; Banissy et al., 2011): namely, they involve supplementary, automatic, involuntary associations between an inducer and a concurrent. In mirror-touch, visual or imaginary stimulation evokes somatosensory experience; in ticker tape, auditory or imaginary stimulation evokes visual experience. However, mirror-touch and ticker tape are minimally idiosyncratic and not arbitrary (Hupé et al., 2012; Rothen and Meier, 2013). Whether these phenomena should be considered a subtype of synesthesia largely depends on the criteria employed but there is preliminary evidence that mirror-touch and synesthesia may co-occur: in a mixed group of systematically-recruited (*n* = 9) and self-referred (*n* = 12) participants, nine (43%) individuals with mirror-touch reported grapheme-personification associations and seven (33%) reported grapheme-color associations (Banissy et al., 2009), well-above the estimates for the general population.

Knowledge of the co-occurrences of mirror-touch and ticker tape with synesthesia could suggest whether these phenomena have similar genetic or neurological underpinnings. As an example, Gregersen et al. (2013) showed that colored-hearing synesthesia was positively associated with absolute pitch (which is not in itself considered a form of synesthesia): out of 768 subjects showing robust evidence of absolute pitch, 20% reported synesthesia, mostly between pitch and color (17% of this population, much higher than estimated in the general population—see Discussion). Combined linkage analysis of multiplex families with synesthesia or absolute pitch suggested that both phenomena were genetically closely related, likely reflecting an underlying commonality of neurodevelopmental mechanisms (Gregersen et al., 2013).

Possible co-occurrence of mirror-touch and ticker tape with synesthesia may, however, be expressed in a complex or subtle manner. Indeed, the very large-scale study by Novich et al. (2011) on about 19,000 self-reported synesthetes suggested that synesthesia may not be a single phenomenon since it appeared to be composed of five independent subgroups: colored sequences, musical colors, colored sensation, non-visual sequelae, and spatial sequence synesthesias. This result could indicate independent neural or genetic mechanisms for these different types of synesthesia (Novich et al., 2011). Co-occurrences of mirror-touch and ticker tape should therefore be searched for at the level of synesthesia subtypes.

The current study had five main goals (1) to examine whether mirror-touch and ticker tape associations are more prevalent in synesthetes than non-synesthetes, (2) to examine whether mirror-touch and ticker tape are associated with specific subtypes of synesthesia, (3) to examine gender differences in the proportions of synesthesia, mirror-touch, and ticker tape experiences, (4) to determine whether proportions of synesthesia, mirror-touch, and ticker tape experiences differ across domain of career and education, and (5) to provide prevalence estimates of phenomenal traits in the French population.

## Methods

### Recruitment

A focal point of this project was its ambition to employ methods for participant recruitment unbiased by self-referral. An effort was made to systematically recruit participants from a large and diverse group. Presentations were given to individuals at eight universities and one museum in Toulouse, southern France, in which a short description of the project was provided (a 5-minute oral presentation in the classroom or a quick explanation of the flyer for the museum). Flyers were then distributed with the internet address for a short online survey, “Interior Experiences.”

This study was conducted across 2 years. The first year involved recruitment at both universities and a museum; due to administrative restraints, recruitment presentations were different for universities and for the general public. In the first year of the study, university presentations included a definition and specific example of synesthesia as one of many different kinds of thought and perception. Flyers given to the general public explained that everyone has a different way of thinking, yet without any reference to synesthesia (note that in France, synesthesia is still unknown by the vast majority of the population, unlike in the United States and the United Kingdom). The proportion of respondents who reported synesthesia was very similar (less than 1% difference) between university and general public samples, so the explicit reference to synesthesia in the first case did not seem to induce more synesthetes to complete the survey. In the second year of the study, only university students were recruited and no reference to synesthesia was made in the presentation. A unique code was given to each person, allowing us to evaluate the response rate for every class and museum group, but the respondents could remain anonymous if desired. Students were recruited from the domains of economics, political science, law, engineering, agronomy, applied science, veterinary, medicine, psychology, and biology. Members of the general public were systematically recruited from conferences at the local Natural History Museum and during city-wide “Brain Week” events. We distributed a total of 3743 flyers.

### Material

#### Interior experiences survey

This 5-minute online survey (whose translation is provided in the Appendix) involved questions concerning general demographic information, career and education, and the following types of synesthesia: grapheme-color (letters and/or numbers evoking colors/forms), temporal-color (numbers and/or time sequences: days, months, centuries, *etc*. evoking colors/forms), sequence-space (numbers and/or time sequences being organized in space), grapheme-personification (letters and/or numbers associated with gender/personality), person-color (colors associated with people), and audition-color/form (sounds/voices/music evoking colors/forms). Since audition-color/form synesthesia may be easily confused with normal multisensory experience, a comment box was provided for explanation and examples of this subtype. An additional “other” comment box was provided (for other multisensory experiences in year one of the study and other types of unique thought/perception in year two of the study), as well as a final comment box where participants were instructed to list any doubts or explanations about earlier questions. We added three more questions in the second year in order to implicate the contribution of those without phenomenal traits (see Results and Appendix).

To assess mirror-touch, participants were asked “When you observe a person being touched on a place on his/her body by someone or something, do you feel the sensation on your own body on the place where the person was touched?” Unlike a previous study that asked participants to rate the degree to which they experience mirror-touch on a five-point scale (Banissy et al., 2009), our participants responded dichotomously (yes/no) and were asked to describe their experiences in a comment box, including whether or not the sensations occurred in a mirrored-fashion (an example was provided). To assess ticker tape, participants were asked two questions: (1) “When you listen to someone speaking, do you automatically visualize the words that he/she is saying (like a “teleprompter” in a way that scrolls in your head)?” and (2) “When you speak (or think verbally), do you automatically visualize the words you are saying?” To reduce the length of the questionnaire, individuals were not asked for a description of their ticker tape experiences, as this phenomenon may be easier to discern than mirror-touch.

### Classification criteria

Consistent with the criteria of synesthesia being arbitrary and idiosyncratic, participants were counted as non-synesthetes if they marked “yes” to questions about synesthesia yet gave only common examples in the audition-color/form, “other,” or final comment box, such as smells triggering tastes or stimulation eliciting emotions and memories: for example, a taste or odor bringing to mind a precise visual memory. Participants were also counted as non-synesthetes if their only descriptions were clearly cultural or metaphorical associations; for example, spring associated with a floral ambiance or red, green, and yellow associated with reggae music. Individuals who gave these types of examples in addition to other valid synesthetic examples were still counted as synesthetes for their other subtypes. Participants were counted as non-mirror-touch if their descriptions only mentioned empathy or emotion without physical experience. Because no specific comment box was provided for ticker tape or other subtypes of synesthesia, anyone who answered “yes” to these questions was counted as a synesthete; furthermore, individuals were classified as ticker tapers whether their visual experiences occurred for words that were heard, spoken/thought verbally, or both.

Individuals' career or education domain was classified into three different groups, according to the French education system: (1) Scientific (S), (2) Economic and Social (ES), and (3) Literary (L). The following career and education areas were coded as Scientific: medicine, veterinary, biology, agronomy, applied science, and engineering. The following areas were coded as Economic and Social: political science, economics, and law. The following areas were coded as Literary: psychology, literature, and language.

### Analyses

*Chi-squared* tests were conducted to examine the following relationships: (1) differences in ticker tape and mirror-touch proportions between groups of self-reported synesthetes and non-synesthetes, (2) differences in ticker tape and mirror-touch proportions across subtypes of synesthesia, (3) differences between men and women in proportions of self-reported ticker tape, mirror-touch, and synesthesia (any synesthesia and across subtypes), and (4) differences among career/education domains in proportions of self-reported ticker tape, mirror-touch, and synesthesia (any synesthesia and across subtypes). Analyses were corrected for multiple comparisons using Bonferroni corrections to maintain 5% family-wise error rates. The pattern of results provoked an investigation of the general tendency to endorse items. To examine this, the relationships among responses to some survey questions were tested *post-hoc*, using point-biserial correlations (Pearson correlations in which one variable is dichotomous) and multiple linear regression.

## Results

### Response rates

Response rates from students and the general population were ~30 and 16%, respectively. Forty-two individuals who began but did not finish the survey and 38 individuals whose maternal language was not French were removed (i.e., not used in the study), providing usable data from a total of 1017 respondents (university: *n* = 900, museum: *n* = 117). Analyses were first performed independently on the data obtained in the 2 years of the study (345 and 672 respondents, respectively). None of the measures appreciably changed between the 2 years so the data were combined.

Of these respondents, ~70% reported at least one type of synesthetic association. Such a high proportion indicates an obvious response bias, as well as potential false-positive reports. We decided to hypothesize a very strong response bias, assuming that those who did not complete the survey had neither synesthesia nor other phenomenal traits. In other words, we considered that all people who thought that their inner experience may be special had the motivation to check the online questionnaire and complete the full survey. Such an assumption is of course very conservative. But without verification using consistency tests, we had no way to detect potential false-reports so our initial criteria were certainly too liberal. We hoped that our conservative assumption would balance our liberal criteria. The comparison of our prevalence estimates with those from the few other studies available (see the Discussion section) indicates that these assumptions put us in the right ballpark.

### Prevalence estimates

Prevalence estimates of phenomenal traits in the population were estimated based on the full recruitment pool receiving flyers (Table 1).

**Table 1:** **Prevalence estimates**.

	**Prevalence estimate (*n* = 3743) (%)**
Any synesthesia (*n* = 712)	19.0
Grapheme-color (*n* = 152)	4.1
Temporal-color (*n* = 268)	7.2
Sequence-space (*n* = 328)	8.8
Grapheme-personification (*n* = 444)	11.9
Person-color (*n* = 245)	6.6
Audition-color (*n* = 169)	4.6
Mirror-touch (*n* = 383)	10.2
Ticker tape (*n* = 260)	6.9

### Co-occurrences (*n* = 1017 respondents)

To examine whether phenomenal traits are more frequent in synesthetes, we computed Pearson χ^2^ values to test whether the co-occurrences of phenomenal traits with subtypes of synesthesia were higher than chance (Table 2, rows 1 and 2). For example, under the assumption of independence between mirror-touch and grapheme-color, we would expect that 57 people with grapheme-color would also have mirror-touch (152 × 383/1017, see Table 1), while the other 95 grapheme-color synesthetes would not experience any mirror-touch. The Pearson χ^2^ value is calculated by comparing the observed values (75 grapheme-color synesthetes who also have mirror-touch and 77 grapheme-color synesthetes without mirror-touch) to these expected values.

**Table 2:** **Co-occurences among subtypes of synesthesia and phenomenal traits, displayed using Pearson χ^2^ values; phi (Φ) effect sizes in parentheses**.

	**Mirror-touch**	**Ticker tape**	**Grapheme-color**	**Temporal sequence-color**	**Sequence-spatial**	**OLP**	**Person-color**	**Audition-color**
Mirror-touch	–	3.8 (0.06)	*10.4 (0.10)*	8.7 (0.09)	*11.5 (0.11)*	*32.7 (0.18)*	*27.6 (0.17)*	*21.2 (0.14)*
Ticker tape		–	8.1 (0.09)	2.4 (0.05)	*18.7 (0.14)*	*12.6 (0.11)*	*15.4 (0.12)*	*10.7 (0.10)*
Grapheme-color			–	***107.5*** ***(0.33)***	*17.1 (0.13)*	*49.4 (0.22)*	*29.4 (0.17)*	*34.1 (0.18)*
Temporal sequence-color				–	*23.1 (0.15)*	*41.7 (0.20)*	*76.1 (0.27)*	*51.8 (0.22)*
Sequence-space					–	*41.8 (0.20)*	*25.2 (0.16)*	*24.4 (0.11)*
OLP						*–*	*54.7 (0.23)*	*37.7 (0.19)*
Person-color							*–*	*66.0 (0.26)*
Audition-color								–

*According to usual conventions (Cohen, 1988), effect sizes can be considered as small (*Φ = *0.10–0.29), medium (*Φ = *0.30–0.49), and large (over 0.5). Effect sizes smaller than 0.10 are likely to reflect spurious correlations and in our study did not reach our statistical criterion correcting for multiple comparisons (Bonferroni correction for 28 analyses: p < 0.0018*, χ^2^ > *9.7. An uncorrected p < 0.05 is obtained for* χ^2^ > *3.84). Small effects are shown in italics. Only one analysis reached a medium effect size, shown in bold and italics.*

Mirror-touch was associated with all six subtypes of synesthesia (association with temporal sequence-color was marginally significant, depending on the level of statistical correction) and ticker tape was associated with every subtype except temporal sequence-color, and only marginally with grapheme-color. Though these associations were significant, the *phi* statistics indicated small effect sizes at best. Considering the correlations with any type of synesthesia, effect sizes were still small (mirror-touch and any synesthesia, χ^2^ = 31.7, Φ = 0.18; ticker tape and any synesthesia, χ^2^ = 13.0, Φ = 0.11). Mirror-touch and ticker tape did not significantly co-occur.

The majority of respondents did not indicate whether their mirror-touch experiences were felt in a mirrored fashion. Of those who did provide this information (*n* = 98), 43% were of the specular (mirrored) subtype and 57% were of the anatomical (non-mirrored) subtype. Similar rates were found among individuals who reported ticker tape experiences for both heard and spoken/verbally thought words (47% of ticker tapers) as for those who reported just one type (53% of ticker tapers). Among those with only one type, it was slightly more common to experience ticker tape for listening (59%) than for speaking/thinking verbally (41%).

We performed similar analyses to evaluate co-occurrences among subtypes of synesthesia (Table 2, rows 3–8). All types of synesthesia were significantly and positively correlated with each other (we observed no co-occurrence lower than chance) but most were at the level of a small effect size. Only the co-occurrence between grapheme-color and temporal sequence-color reached the level of a medium effect size.

### Career/education domain (*n* = 1,017 respondents)

In the first year of the study, we compared ES, S, and L domains. We found no differences among the three domains. In the second year of the study, recruitment was only conducted at S and ES universities. We summed the responses across both years using the common S (*n* = 526) and ES (*n* = 368) domains and found no significant difference for mirror-touch (χ^2^ = 0.9, *p* = 0.34), ticker tape (χ^2^ = 0.13, *p* = 0.72), or synesthetic subtypes (χ^2^ values ranged from 0.001 to 4.0, all *p* > 0.047, uncorrected).

### Gender comparisons (*n* = 1,017 respondents)

No significant differences were found between men (*n* = 321) and women (*n* = 696) for rates of mirror-touch (χ^2^ = 2.7, *p* = 0.10), ticker tape (χ^2^ = 1.2, *p* = 0.28), or synesthetic subtypes (χ^2^ values ranged from 0.23 to 2.6, all *p* > 0.08, uncorrected), when summing across both years of the study. The same pattern of results was found in both years of the study. Note that more women filled out the online questionnaire than men. However, we do not know whether this difference reflects a response bias or a sampling bias, since we do not know the male/female ratio of the population to which we distributed the flyers.

### Acquiescence

Our pattern of results (significant, positive correlations between most items) suggested that some individuals might be more likely to endorse items in general. In order to evaluate possible acquiescence effects, we conducted *post-hoc* analyses to examine responses to three items unrelated to the study. Note that these questions were not originally designed for the purpose of examining acquiescence but were added during the second year of the survey so that individuals without phenomenal traits would still feel implicated: “How often do you remember your dreams?” (always, often, sometimes, never/rarely), “Do you have memories before the age of 5?” (yes, no, I don't know), “How often do you have a song stuck in your head?” (often, sometimes, never/rarely). The question on dreams was scored from 1 to 4 and the question on songs was scored from 1 to 3. The question on memories was scored dichotomously, with a response of “I don't know” scored as zero, representing a lack of acquiescence. It is unknown whether these items might be related to phenomenal traits: having a song stuck in one's head could presumably be associated with subtypes of synesthesia that have auditory inducers but there is no strong argument for the other questions being associated with phenomenal traits. Therefore, the correlations of these items with mirror-touch, ticker tape, any synesthesia, and the six subtypes of synesthesia were examined for possible effects of acquiescence.

Twenty-seven point-biserial correlations were conducted so the family-wise error rate was set to *p* < 0.0019. The frequency of remembering one's dreams correlated significantly with global synesthesia (*r*^2^ = 0.017), sequence-space (*r*^2^ = 0.026), and OLP (*r*^2^ = 0.014). A multiple linear regression analysis showed that global synesthesia did not explain any meaningful variance in the endorsement of remembering one's dreams, over-and-above that explained by sequence-space and OLP (*r*^2^ change = 0.001, *F* change = 0.61, *ns*), suggesting that this correlation was specific to the two subtypes. Though significant, the correlations were well-below the usual criterion to even qualify as weak (see Cohen's criteria for effect sizes in the legend of Table 2). The four other subtypes of synesthesia and phenomenal traits were unrelated to general items. Moreover, the weak correlation for sequence-space and OLP was only found for one out of the three questions. Individuals with synesthesia, mirror-touch, and ticker tape are in fact not more likely to acquiesce on the majority of general items. The relations within subtypes of synesthesia and to phenomenal traits were therefore unlikely due to over-endorsement of items.

## Discussion

Few systematic studies exist to date on the prevalence of synesthesia, certain synesthetic subtypes, and mirror-touch; to our knowledge, no previous study has tried to evaluate the prevalence of ticker tape. Knowledge of the frequency of synesthesia and phenomenal traits is important both for informing the general public and to guide future research efforts (e.g., sample size and recruitment requirements). High prevalence rates of certain subtypes may also be a concern for studies not interested in synesthesia a priori but in general cognitive traits, since undisclosed synesthetic experiences may interfere with other measures, as in the example of sequence-space synesthesia (e.g., Price and Mentzoni, 2008; Price and Mattingley, 2013, for a review) for the SNARC effect (spatial-numerical association of response codes; Dehaene et al., 1993). Synesthetic associations between letters and colors may also promote cognitive and memorization strategies and bias the results of certain tests (Rothen et al., 2012). The presence (or absence) of co-occurrences between subtypes of synesthesia and phenomenal traits may suggest possible common (or independent) genetic origins and neuronal mechanisms for the development and expression of these traits. The current study brings new information to these questions, though exact figures should be interpreted with caution due to methodological limitations. In this discussion, we will weigh the arguments for and against the validity of our findings.

Important limitations of our study are the brevity of the screening questionnaire and the absence of verification using consistency tests, so we had no way to detect potential false-reports. However, the free reports provided in the comment boxes, as well as a follow-up study with a subset of participants recruited from this screening (Chun and Hupé, 2013 [Abstract]; see Anecdotal Reports, below) yielded rich information, supporting the valid recruitment of authentic synesthetes.

Another strong limitation of our study is that less than a third of the people to whom we distributed flyers filled out the online questionnaire. The very high prevalence rate of synesthesia that we measured among those who did respond suggested a strong bias presiding upon the choice to fill out the questionnaire. Our prevalence numbers (Table 1) are based on the hypothesis of this strong response bias, assuming that those who did not complete the survey had neither synesthesia nor other phenomenal traits. This hypothesis is obviously too conservative, but it seemed to balance out our overly liberal inclusion criteria (without verification of experiences). Indeed, when comparing our estimated prevalence rates with those obtained with stronger methodology, when available, we found in most cases a similar order of magnitude (see Prevalence Comparisons, below). This allows us to hypothesize that our relative rates for subtypes of synesthesia are fairly accurate and our novel prevalence rates provide an adequate first approximation.

Our measures of co-occurrences between subtypes of synesthesia and phenomenal traits could also be contaminated by response bias, if people with some specific traits were for any reason more (or less) motivated to fill out the online questionnaire. Without completely ruling out this possibility, several observations argue for a limited influence of such a bias. First, we measured similar rates of synesthesia and phenomenal traits in men and women. Previous gender differences reported in synesthesia (e.g., Baron-Cohen et al., 1993) are now thought to be due to disparity in self-disclosure (Ward and Simner, 2005). The finding of equal gender proportions in the current study thus diminishes the likelihood of self-disclosure biases in our sample, as equal rates of synesthesia in males and females were found in large-scale studies that verified authentic associations in systematically recruited samples (Sagiv et al., 2006; Simner et al., 2006) and a mixed systematic and self-referred sample (Seron et al., 1992). A second, incidental validation of our recruitment method was provided by the results of year one. As indicated in the Methods section, the University and Museum groups received different instructions, with reference to synesthesia only in the first group. Yet the results were highly similar in both groups, suggesting that the response bias of completing the survey was not specific to synesthesia. A third argument in favor of the validity of our results on co-occurrence comes from the comparison with the few numbers available in the literature, based either on systematic recruitment or large-scale self-reports (see *Co-occurrence Comparisons*, below).

### Anecdotal reports

There was considerable variety in individuals' experience of phenomenal traits. Mirror-touch was described for many different sensations, including: pain, general pleasure, sexual pleasure, kissing, temperature, tickling, pinches, etc. We even received reports of mirror-touch experiences in response to observation of very specific activities, such as clipping fingernails or putting on lotion. This is consistent with reports that mere observation or imagination of motor activity can induce synesthetic associations, as seen in swimming-style synesthesia (Nikolic et al., 2011; Mroczko-Wasowicz and Werning, 2012). Almost all reports of mirror-touch described direct reciprocation of the localization of touch (whether specular or anatomical). We received less common reports from people (*n* = 3) who always experienced tactile perceptions in the same place, regardless of localization of observed touch; for example, “the inner thigh,” “the spinal cord,” or “a shiver of pain that scrapes from the left armpit to the forearm.” Intensity of perception was also differentially experienced: some reported that observed pain was directly related to perceived pain, even to the point that it became “handicapping and unbearable.” For others, perceived intensity was more or less independent from the strength of observed pain, felt as more of a tightening or a twinge.

Banissy et al. (2009) previously reported that almost 20% of individuals with mirror-touch also experienced personal tactile sensations when observing a lamp being touched. Three participants in our study (two grapheme-color synesthetes and one number-space synesthete) reported similar object-tactile associations, in which someone touching their personal belongings led to experience of touch on their own body (e.g., “a prickling sensation on the back of my neck that is both painful and pleasurable”). Note that these participants offered this information in a comment box even though they were not directly asked about these perceptions, so the occurrence is undoubtedly higher than what we found. Unlike perceptions in response to lamps (Banissy et al., 2009), each of our three participants reported this experience specifically for their personal possessions. This suggests that emotion may play a role in the experience of tactile phenomena such as mirror-touch and synesthesia. While some participants' tactile experiences generalized to strangers and fictional characters, many reported that their mirror-touch responses were enhanced for—or even limited to—people with whom they feel close. Such reports are consistent with previous findings, like those showing that mirror-touch perceptions are stronger for observed touch of real bodies than of dummy bodies (*n* = 14; Holle et al., 2013).

Ticker tape experiences also showed a wealth of individual differences, as reported in semi-structured interviews of participants recruited from our sample for another study (Chun and Hupé, 2013 [Abstract]; ticker tape: 7 men, 11 women). Most participants reported a constant size and font for visualized letters; however, some individuals reported experiencing a change in letter size depending on the volume with which words are spoken. The way in which ticker tape perceptions were “displayed” varied as well: we received reports of both static display, on a screen inside the head or in front of the body, and dynamic display, with words that stream out through the mouth or from behind the head. One ticker taper reported that during a verbal fluency task, ideas “stacked up” visually behind her head before streaming through her mouth as she said them aloud. When too many ideas were being held there, some would disappear before she could say them and thus disappeared from memory. A subset of ticker tapers described visualizing noises spelled out onomatopoetically (“as in a comic book”), whereas others did not. Likewise, some ticker tapers reported spelling out words phonetically from an unknown language while for others, ticker tape seemed directly linked to comprehension: they reported that hearing a language they do not understand would fail to elicit ticker tape.

### Prevalence comparisons

Table 3 shows a comparison of prevalence estimates between the current study—employing systematic recruitment without verification of associations—and previous systematic recruitment studies that were able to verify subjects' associations. Due to the use of different populations, different recruitment and sampling strategies, and different diagnostic criteria among studies, their comparability is arguably limited. However, prevalence estimates in the current study are not significantly different from those previously reported in the literature for grapheme-color^2^ and sequence-space associations, as well as for initial self-report of mirror-touch. Our estimates are slightly higher than previous reports for person-color and temporal sequence-color and are much higher than previous prevalence estimates for OLP; hypotheses to explain such discrepancies are proposed below. Though the estimated prevalence of audition-color in the current study appears elevated compared to a previous report, this difference could be due to the questions we asked (see Appendix, Interior Experiences Survey): we asked participants whether they associated colors with sounds and voices, in addition to music (Simner et al., 2006).

**Table 3:**
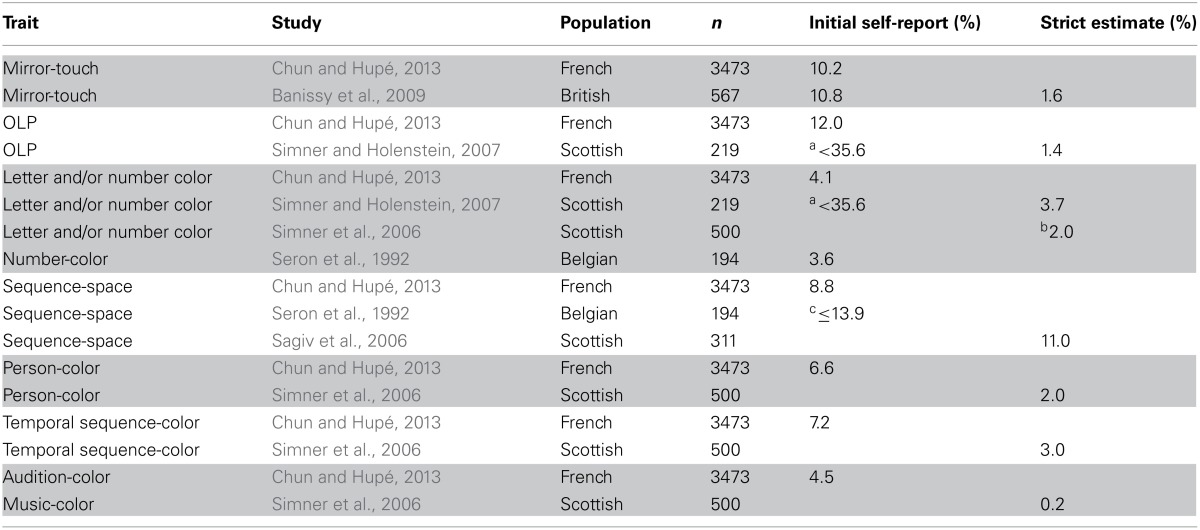
**Prevalence comparisons**.

^a^Simner and Holenstein, 2007: 78 out of 219 individuals initially reported “some type of OLP and/or grapheme-color synesthesia.” From this group, 21 (9.6%) scored higher than controls (2–3 week retest) 5 weeks later and 10 (4.6%) continued to score higher 1 year later. The strict estimates are derived from these 10 individuals: 2 with OLP, 7 with grapheme-color, and 1 with both.

^b^Simner et al., 2006: For comparability with the current study, the proportion of individuals with letter- and/or number-color associations are reported from Simner et al.'s data; therefore this figure is slightly different than the 1.4% typically cited from the university study, referring to individuals with both letter- and number-color associations.

^c^Seron et al., 1992: 194 individuals were recruited systematically to take a brief questionnaire. From this group, 27 individuals gave a positive response regarding “some particular number representation;” no breakdown was provided for group composition from the three measured types of associations: sequence-space, number-color/form, and simple analogical representations (“the quantity was directly represented by patterns of dots or other things such as alignment of apples, parts of a bar of chocolate, etc.”). An additional non-systematic, informal inquiry yielded 22 more positive responses. From the mixed group of 49 individuals, 26 agreed to answer a more detailed questionnaire; however, it is unknown how many of these verified associations came from the systematically-recruited group. The sequence-space prevalence is therefore less than or equal to 27/194. It should be noted that sequence-space composed 74% of positive responses from the detailed questionnaire and 68% of positive responses from the brief questionnaire. If the frequency found from the detailed questionnaire is accurate, one might speculate the sequence-space prevalence to be ~10% (0.74 × 13.9%).

#### Phenomenal traits

To our knowledge, this is the first study to present systematic data on ticker tape experiences. Prevalence rates are estimated at 7% for ticker tape and 10% for mirror-touch. In a previous mirror-touch study, detailed interview and examination of response to videos of tactile stimuli reduced the number of potential mirror-touch subjects by a factor of over 4 (Banissy et al., 2009). Though specific elimination criteria were not provided, this yields two possible implications for the current study: (1) genuine mirror-touch is consistent and our prevalence estimate is too high, or (2) mirror-touch lacks the consistency of synesthesia (Rothen and Meier, 2013).

In contrast with the previously found preponderance of specular mapping in individuals with mirror-touch (*n* = 17 specular vs. 3 anatomical; Banissy et al., 2009), the current study found relatively equal rates of mirror-touch subtypes, favoring anatomical mapping (*n* = 42 specular vs. 56 anatomical). Though this large disparity in subtypes could mean that anatomical mapping is more prevalent in the French population, it seems more likely that the associations of those reporting anatomical mapping have lower consistency, as they were less frequently identified with the use of stringent criteria (Banissy et al., 2009).

#### Ordinal-linguistic personification

OLP synesthesia may be more prevalent in Francophone (12%) than in Anglophone (1.4%) populations. This would be logical given the masculine-feminine categorization built into the structure of the French language. In French, grammatical gender exists only for words (which we did not specifically inquire about) but personification associations are seen at the level of numbers and letters. It has already been shown that childhood cultural experience can shape the expression of specific associations within synesthesia (Ward and Simner, 2003) but it is an empirical question whether culture and/or maternal language may affect the actual development and prevalence of synesthesia within a population. The idea that grammatical gender may shape thought specifically related to personification attribution has already been proposed (Amin et al., 2011).

The potential role of culture and maternal language on the development and expression of synesthesia remains speculative for several reasons: (1) the current study lacked verification of associations, (2) Simner and Holenstein's (2007)'s study may have had an insufficient sample size to make a stable prevalence estimate (3 synesthetes from a group of 219), and (3) Simner and Holenstein (2007) used a very conservative procedure (see Table 3, footnote 1) aimed at specifying the lower bound of this estimate.

#### Person-color

One possible cause of the discrepancy in observed prevalence rates for person-color associations (6.6% in our study vs. 2% by Simner et al., 2006) could be related to cultural differences in the desire to conceal these associations, due to the stigma related to mystical aura-reading. Non-idiographic, synesthetic-like person-color associations (i.e., associating a person with a frequently-worn color or with a physical attribute, such as hair/eye color) may be more common than synesthetic-like associations for other subtypes, such as grapheme-color; therefore it is also possible that these non-idiographic associations were more easily identified and eliminated with face-to-face screening compared with online screening.

### Co-occurrence comparisons

Table 4 shows a comparison of co-occurrence rates between the current study and previous studies that used at least partial systematic recruitment. The same general trends in co-occurrence patterns lend validity to the current examination. Banissy et al. (2009) observed a high incidence of both grapheme-color and grapheme-personifications in their small sample of verified mirror-touch individuals, indeed suggesting co-occurrence of mirror-touch with synesthesia. Simner et al. (2006)'s systematic examination showed that grapheme-color and temporal sequence-color were highly correlated, in agreement with our largest observed effect size. Unlike what was found in the current study, however, they found grapheme-color and temporal sequence-color to be completely independent from person-color and audition-color, with zero cases of co-occurrence.

**Table 4:**
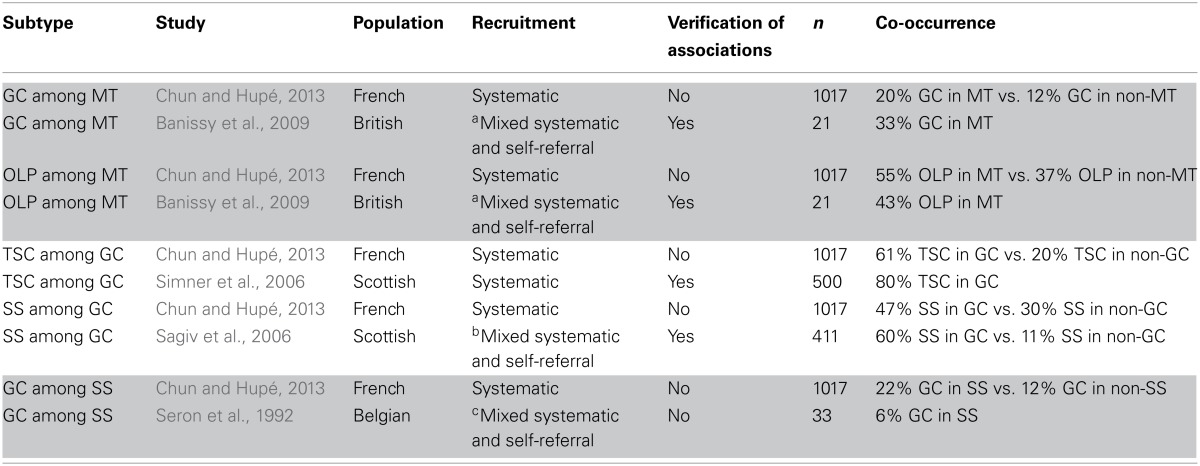
**Co-occurrence comparisons**.

GC, Grapheme-color; MT, Mirror-touch; OLP, Ordinal-linguistic personification; SS, sequence space; TSC, Temporal sequence-color.

^a^Banissy et al., 2009: 9 individuals were recruited systematically and 12 individuals were recruited by self-referral.

^b^Sagiv et al., 2006: Non-grapheme-color synesthetes were recruited systematically (n = 311) but grapheme-color synesthetes (n = 100) were self-referred online.

^c^Seron et al., 1992: From a mixed recruitment group (see Table 3, footnote 2 for a full explanation), detailed questionnaires showed 1 out of 20 SS who had GC as well; brief questionnaires showed 1 out of 13 SS who had GC as well.

Sagiv et al. (2006) examined the occurrence of number forms in both grapheme-color synesthetes and non-synesthetes (that is, not including number forms in the definition of synesthesia). They found a higher proportion of number form cases in grapheme-color synesthetes. The greater rate of co-occurrence found in their study compared to our study could be due to their different recruitment procedures for grapheme-color synesthetes (no systematic recruitment) and non-grapheme-color synesthetes (systematic recruitment). Seron et al. (1992) reported the number of grapheme-color synesthetes among individuals with sequence-space. This time the number of co-occurrences was lower than observed in our study but here as well, recruitment was not homogeneous. Simner and Holenstein (2007) measured both grapheme-color and OLP, but their strict criterion for inclusion restricted their sample to only three people with OLP (see Table 3, footnote 1), precluding meaningful statistical comparisons.

Novich et al. (2011) conducted the largest study to date on co-occurrences between subtypes of synesthesia, on the basis of about 19,000 self-referred reports. However, like in our study, most subtypes could not be verified. Prevalence estimates were not possible since only potential synesthetes filled out their online questionnaire. Relative prevalence rates of the different subtypes were also not possible to calculate, since grapheme-color synesthetes were apparently more motivated to visit the “synaesthesia battery” website (probably due to research interests and media coverage). This bias is expressed in their high proportion of grapheme-color synesthetes (about 40%) compared to sequence-space synesthetes (31%), while systematic recruitment studies have found a much higher prevalence of sequence-space than grapheme-color, comparing both within (Seron et al., 1992) and across populations (i.e., Sagiv et al., 2006 vs. Simner et al., 2006). This strong bias means that their observed rates of co-occurrences could not be extrapolated to the general population, as demonstrated by the following thought experiment: if only grapheme-color synesthetes visited the synaesthesia battery website, then all sequence-space synesthetes would also report grapheme-color synesthesia. In spite of such a bias, the main result of that study—a clustering of subtypes of synesthesia—is probably valid, and in that case very informative. Continuing the thought experiment, if only grapheme-color synesthetes visited the synaesthesia battery website, that alone would not lead to a higher proportion of those also experiencing colors for temporal sequences than those also experiencing sequence-space (as observed by Novich et al., 2011). Such strong bias would predict the same proportion of grapheme-color synesthetes (that is, 100% in this extreme case) among their whole sample and the subset of synesthetes with sequence-space (as observed by Novich et al.), but with no influence on the proportions of synesthetes with sound-color associations, for example, in the whole sample and among sequence-space synesthetes. Therefore we have no reason to suspect that their recruitment bias questions their observed clustering of subtypes of synesthesia within five groups. Such clustering leads to precise predictions for our study. Among the five subtypes included in both Novich and our study, four types belonged to different groups. Only grapheme-color and temporal sequence-color belonged to the same group. In agreement with Novich et al. (2011), co-occurrence between these two types was the only one in our study that reached a medium effect size.

Novich and colleagues emphasized the relative independence between subtypes of synesthesia, showing, for example that the proportion of people having each type of synesthesia was very similar for synesthetes with or without sequence-space synesthesia. Our results do not contradict this observation: sequence-space synesthesia was significantly correlated with every other subtype, not any subtype in particular (all small effect sizes, *phi* between 0.11 and 0.20—see Table 2). Novich and colleagues could not measure such a correlation because they had no control group without synesthesia.

Our results therefore show that, even if synesthetic subtypes cluster in different groups, as shown by Novich et al. (2011), synesthetes tend to experience several subtypes of synesthesia, an important argument for inclusion within a unique phenotype. Following such logic, one may argue for including mirror-touch and ticker tape also within the synesthesia phenotype. However, co-occurrence should not be the sole criterion considered, as exemplified by the co-occurrence of absolute pitch and synesthesia (Gregersen et al., 2013). Moreover, the average effect sizes of co-occurrences between phenomenal traits and synesthesia were weak (0.13 for mirror-touch and 0.10 for ticker tape), even weaker than between subgroups of synesthesia (0.19). Given the high uncertainty surrounding these numbers (due to our methodological limitations), further research will be necessary before reaching any strong conclusion. At this stage, we would like to conclude that genetic and/or neurological links between synesthesia, mirror-touch and (but to a lesser degree) ticker tape, are plausible.

## Conclusions

Our study had five main goals. First, to examine whether mirror-touch and ticker tape associations are more prevalent in synesthetes than non-synesthetes. The answer is yes (which may indicate common genetic or neural mechanisms), though only to a weak degree in our study, and we cannot exclude that the elevated frequency of these phenomenal traits in synesthetes resulted from our recruitment bias. Our second goal was to examine whether mirror-touch and ticker tape are associated with specific subtypes of synesthesia. The answer is no: co-occurrences, if real, were distributed across all subtypes. Our third aim was to examine gender differences in proportions of synesthesia, mirror-touch, and ticker tape experiences; no differences were found. The fourth goal was to determine whether proportions of synesthesia, mirror-touch, and ticker tape experiences differ across domain of career and education; no differences were found. Finally, we aimed to provide prevalence estimates of phenomenal traits in the French population. We estimated ticker tape at 7% and mirror-touch at 10%. These numbers place the prevalence of these phenomena within the range of those of grapheme-color (4%) and sequence-space (9%), the most studied subtypes of synesthesia. We observed frequent associations of people with colors (7%) and graphemes with gender or personality (12%). These proportions are higher than previously presumed, based indirectly on sampling of Anglo-Saxon populations. We suggest that grapheme-personifications may be more frequent in the French population. If confirmed, this cultural difference would show that culture and maternal language play an important role in the development and/or expression of synesthesia.

The main strength of this study was its systematic recruitment, though the sample was still biased toward scholarly individuals. The use of a brief online questionnaire yielded a sizeable sample but introduced greater ambiguity than face-to-face studies. The study's main limitation was our inability to test the authenticity and consistency of participants' perceptions. For example, report of synesthetic-like experiences resulting from drug use or neurological conditions reflect possible sources of error. In light of these limitations, the authors made every effort to provide conservative estimates of synesthesia and phenomenal traits. However, without verification of the consistency and number of synesthetic associations, the group of synesthetes may be better described as “individuals with synesthetic-like experiences.” Considering these shortcomings, evidence of a higher prevalence of mirror-touch and ticker tape associations in the synesthetic population is tentative.

### Conflict of interest statement

The authors declare that the research was conducted in the absence of any commercial or financial relationships that could be construed as a potential conflict of interest.

## Footnotes

^1^Galton F. Inquiries into human faculty and its development. London: MacMillan, 1883, p. 67: “Some few persons see mentally in print every word that is uttered; they attend to the visual equivalent and not to the sound of the words, and they read them off usually as from a long imaginary strip of paper, such as is unwound from telegraphic instruments. The experiences differ in detail as to size and kind of type, colour of paper, and so forth, but are always the same in the same person.”

^2^When combining the results of both studies by Simner and colleagues that reported the proportions for color associations to letters or numbers, the proportion of synesthetes was 18/719 (= 2.5%), which is only marginally different from 4.1% (152 synesthetes among 3473 individuals; χ^2^ = 3.99, *p* = 0.046).

## Appendix

### Interior experiences survey

*Author's Note:* The survey questions were completed in sequential order, such that subsequent questions were not visible until the previous question had been filled out.

#### (A) Introduction

Thank you very much for accepting to participate in this study, led by the Brain and Cognition Research Center, laboratory of the University of Toulouse and of CNRS (http://cerco.ups-tlse.fr). Please respond to all the questions below concerning your activities and your personal experiences, as well as some general information. It shouldn't take much more than 5 minutes.

Know that your responses and personal information—if you provide us with them—will be kept confidential and evidently won't be distributed to exterior parties. You will have the possibility to give us your email address at the end of the questionnaire. In this case, we will be able to ask you to participate in the next step of our study. We will select a varied sample, representative from this questionnaire if possible. So it is important that you complete it carefully even if you do not wish to participate in the next step of the study.

If you are willing and selected, we will later ask you to respond to other questions (via internet), then to come in to the laboratory to take several playful tests, using your creative and imaginative capacities. We will then explain in more detail what that consists of and of course you will always have the choice to continue or stop your participation at any moment. You will be compensated for the time spent at the lab.

If you have questions, do not hesitate to contact us.

#### (B) General information

1. What is the code indicated on the piece of paper we distributed to you?
2. First and last name (or your initials if you do not want to participate in the rest of the study—see below).
3. Birthday
4. Where do you live? (city and department)
5. Maternal language
6. Are you multilingual? (considered as any language in which you think—habitually or in certain contexts)
  Yes/No
  If yes, indicate which languages you learned before starting school
7. Gender
  Male/Female
8. Handedness
  Left/Right/Ambidextrous
9. Main professional activity (student is a possible answer)
10. Highest level of education or diploma obtained to date
11. Main specialization of your studies

#### (C) Your activities

1. Do you have a regular artistic activity?
  (We consider all types of artistic practices—whether in fine arts, photography, music, dance, theatre, writing, etc.—from the moment that you produce a “piece or work,” whether in an institutional or private setting).
  Yes/No
  If yes, explain which type and an approximate frequency
  If yes, do you ever present your pieces in public (or have representations in public)?

#### (D) Your subjective experiences

It's possible that certain questions are difficult to understand—in this case, there is a chance that it concerns a particularity that you don't have and you should answer “no” to the question. If you have doubts, you can indicate and explain them in the space provided for this later.

1. When you think, would you say that it's in verbal form (with an interior dialog)?
  Always/Often/Sometimes/Very rarely or never
2. When you think, would you say that it's in the form of images?
  Always/Often/Sometimes/Very rarely or never
  If this happens, can you explain the dominant nature of these images? (for example, purely visual, auditory, olfactory, audio-visual, etc.)
^B^3. Do you remember your dreams?
  Always/Often/Sometimes/Very rarely or never
^B^4. Do you have memories before the age of 5?
  Yes/No/I don't know
  You may explain your earliest memory, if you would like.
^B^5. Do you get a song stuck “on repeat” in your head?
  Often/Sometimes/Very rarely or never
6. When you listen to someone speaking, do you automatically visualize the words the person is saying (like a “prompter” in a way, that scrolls through your head)?
  Yes/No
7. When you speak (or think verbally), do you automatically visualize the words you are saying?
  Yes/No
8. When you observe a person who is touched on a place on their body by something or someone, does it happen that you feel the sensation on your own body in the place the person was touched?
  Yes/No
  If yes, explain whether it is systematic. Indicate whether your sensation is mirrored (for example, if a person across from you is touched on their right arm—that is therefore on your left side—do you feel the sensation in your own right arm or your left arm, therefore mirrored). If your experience is close to this but is different than what is described, please explain as well.
9. Do you associate letters or numbers with specific colors?
  Yes/No
10. Do you associate temporal sequences (like days of the week or months of the year) with specific colors?
  Yes/No
11. Do numbers or temporal sequences have a particular spatial organization for you?
  Yes/No
12. Do letters or numbers have a gender for you: masculine/feminine?
  Yes/No
13. Do letters or numbers have a specific personality for you?
  Yes/No
14. Do you associate specific colors to people? Yes/No
15. Are there sounds (like voices or music) that systematically evoke colors or specific forms for you?
  Yes/No
  If yes, please explain (examples are welcome)
^A^16. Does stimulation in another sensory modality evoke a sensation or strong association in another modality (vision, audition, touch, odor, taste, movement, emotion)? (other associations than those of audiovisual asked in the previous questions)
^*B^17. Does touch systematically evoke color or forms for you?
  Yes/No
  If yes, please explain (examples are welcome)
^A^18. Do you know or think you have other immediate family members (siblings, parents, children, cousins, nieces/nephews, or aunts and uncles) that would have responded yes to one of the questions in this section?
  If yes, please explain and tell how many (number of brothers, sisters, etc.)
^A^19. How many immediate family members do you have?
  Explain (number of brothers, sisters, etc.)
^B^20. Do you have other ways of thinking or perceiving that you find are different from most of your friends and family?
  Yes/Not to my knowledge
  If yes, please explain (examples are welcome)
  If you responded “yes” to at least one of the questions 9–17, you are most likely what we call a “synesthete.” You can find more information at the following internet address:
  http://cerco.ups-tlse.fr/~hupe/synesthesie.html
  If you have doubts or explanations to give, please indicate them here, with the number to which they correspond.
21. Are you willing to be recontacted for the next step of this study? (We will explain what that consists of in more detail and then you may decide whether or not to participate).
  Yes/No
22. Your email address:
  Thank you for your time and participation! You may find the description of our research project et follow its development at:
  http://cerco.ups-tlse.fr/~hupe/experienceinterieure.html

## Author's Note:

^A^Only asked in year one of the study

^B^Only asked in year two of the study

^*^Touch-color experiences were asked about in the second year of the study as part of a recruitment effort for interview-based research on touch-color and orgasm-color synesthesia. It was not included in the current analyses due to its different sample size, as well as the already large number of variables and time constraints in the current study.
